# Role of arterial blood gas (ABG) as a valuable assessment tool of disease severity in SARS-CoV-2 patients

**DOI:** 10.5937/jomb0-30927

**Published:** 2022-02-02

**Authors:** Jyot Amrita, Arvinder Pal Singh

**Affiliations:** 1 Sri Guru Ram Das University of Health Sciences, Department of Biochemistry, Amritsar, Punjab, India; 2 Sri Guru Ram Das University of Health Sciences, Department of Anesthesia, Amritsar, Punjab, India

**Keywords:** arterial blood gas, SARS-CoV-2, ionized calcium, hypocalcemia, arterijski gasovi krvi, SARS-CoV-2, jonizovani kalcijum, hipokalcijemija

## Abstract

**Background:**

COVID-19 is caused by a novel coronavirus, named severe acute respiratory syndrome coronavirus 2 (SARS-CoV-2). The foremost predominant complication of SARS-CoV-2 is arterial hypoxemia thereby disturbing lung compliance, requiring mechanical ventilation. The aim of the current research study is to analyze role of ABG as a valuable assessment tool of disease severity in SARS-CoV-2 patients.

**Methods:**

170 arterial blood samples were collected from patients admitted in Intensive Care Unit (ICU) of Sri Guru Ram Das Charitable Hospital, Amritsar. They were analyzed for arterial blood gas using ABG analyzer. Parameters of ABG such as pH, pCO2, HCO3, O2 saturation, ionized calcium (iCa) and calculated ionized calcium (at pH 7.4) was calculated for all the samples.

**Results:**

Continuous variables were described as medians with interquartile ranges (IQRs) and categorical variables as percentages and frequencies. Spearman correlation test was done for calculation of correlation between pH and other ABG parameters. Analysis of arterial blood gas revealed significant negative correlation (p<0.05) between pH and pCO2 and significant positive correlation (p<0.05) between pH and HCO3 and between pH and delta ionized calcium. Low levels (98.2%) of ionized calcium were observed while monitoring the ABG findings though weak negative correlation (p<0.05) was observed between pH and iCa.

**Conclusions:**

Our study suggests that ABG analysis acts as a momentous indicator for critically ill patients admitted in Intensive Care Unit (ICU). Estimation of iCa in this critical care setting acts as a distinctive biochemical feature of SARS-CoV-2 disease, as an initial assessment tool, for hypocalcemia.

## Abstract (sr)

**Uvod:**

COVID-19 je izazvan novim koronavirusom koji izaziva težak respiratorni sindrom koronavirus 2 (SARS-CoV-2). Predominantna komplikacija SARS-CoV-2 je arterijska hipoksemija koja dovodi do oštećenja pluća i zahteva mehaničku ventilaciju. Svrha ovog izučavanja je da analizira ulogu AGK kao vrednog parametra za procenu težine oboljenja kod SARS-CoV-2 pacijenata.

**Metode:**

Uzeto je 170 arterijskih uzoraka krvi od pacijenata koji su bili primljeni u Intenzivnu jedinicu (ICU) u Sri Guru Ram Das Charitable Hospital, Amristar. Analizirani su arterijski gasovi krvi primenom ABG analizatora. Parametri ABG (AGK) bili su pH, pCO2, HCO3, O2 saturacija, jonizovani kalcijum (iCA) i izračunati jonizovani kalcijum (na pH 7,4) za sve analizirane uzorke.

**Rezultati:**

Za sve promenljive parametre izračunata je medijana i interkvartilne oblasti (IQRs) i varijable kako što su procenti i frekvencije. Korišćen je Spearman korelacioni test za izračunavanje korelacije između pH i i drigih ABG parametara. Analizom arterijskih gasova krvi dobijena je značajna negativna korelacija (p <0,05) između pH i pCO2 i značajna pozitivna korelacija (p<0,05) između pH i HCO3 i između pH i delta jonizovanog kalcijuma. Niski nivoi (98,2%) jonizovanog kalcijuma su nađeni pri praćenju ABG nalaza u toku nedeljne negativne korelacije (p<0,05) kad je praćena između pH i iCa.

**Zaključak:**

Naše izučavanje ukazuje da ABG analiza ima ulogu trenutnog indikatora za kritično bolesne pacijente primljene u Intenzivnu Jedinicu (ICU). Određivanje iCa u ovom kritičnom momentui ima veoma značajnu ulogu u praćenju SARS CoV-2 oboljenja kao inicijalno sredstvo ispitivanja hipokalcijemije.

## Introduction

The outbreak of Novel coronavirus disease (COVID-19) was initially noticed in a seafood market in Wuhan city in Hubei Province in Mid-December, 2019 and subsequently speeded to 214 countries worldwide [1]. World Health Organization (WHO) declared this outbreak as a »Public Health Emergency of International Concern (PHEIC) on 30^th^ January, 2020 and later declared COVID-19 as pandemic on 11^th^ March, 2020 [2].

The novel coronavirus belongs to lineage B of the genus beta - coronavirus of the coronavirus family which includes SARS - CoV (Severe Acute Respiratory syndrome) and MERS - CoV (Middle East Respiratory Syndrome) [3]. The seventh member of the corona virus family to infect humans is 2019 novel coronavirus (2019-nCoV) [4]. Coronaviruses (CoVs) are positive sense single stranded RNA viruses of the family coronaviridae that infect a wide host range to produce disease ranging from common cold to severe illness [5].

SARS-CoV2 affects different people in various ways. Most infected people develop mild to moderate illness and may recover without hospitalization. Primarily transmitted through the respiratory tract, the most common clinical presentations of symptomatic individuals infected with SARS-CoV-2 include fever, dyspnea, cough, fatigue, and sore throat. In advanced cases, patients may rapidly develop respiratory failure with acute respiratory distress syndrome, and even progress to death [6].

The foremost predominant complication of SARS-CoV-2 is arterial hypoxemia thereby disturbing lung compliance requiring mechanical ventilation. Arterial blood gas (ABG) analysis provides information regarding patient oxygenation, ventilation adequacy and acid base levels.

Or in other words we can say, it tells the activity in both respiratory system and metabolic system. Both the systems work together in order to maintain the pH in normal range. If one system is disturbed the other will try to compensate. Thus, the current research was aimed to analyze the role of ABG as a valuable assessment tool of disease severity in SARS-CoV2 patients. We also hypothesized that which parameter among ABG analysis can play a significant role and to what extent as regards to disease severity.

## Material and Methods

### Participants

In the present observational study 170 samples of ABG were collected from 17 critically ill patients severely affected with the disease and admitted in Intensive Care Unit (ICU) of Sri Guru Ram Das Charitable Hospital, Amritsar, Punjab (India) during the period from October 2020 to December 2020. They were laboratory confirmed tested positive cases for corona virus. The diagnosis was established by reverse-transcriptase polymerase chain reaction (RT-PCR) method by testing nasal and pharyngeal swab specimens according to World Health Organization (WHO) interim guidance criteria. Hematology testing was conducted on H560 Hematology analyzer (Erba Mannheim). Analysis of ABG was done on ABG analyzer (Siemens make) in clinical laboratory of the hospital for arterial samples of all admitted patients whose stay was more than 5 days. The clinical outcome was monitored until the discharge of the patient. No written informed consent of patient was required as the study included only undisclosed information. The study was approved by the institutional ethics committee.

Before proceeding with the analysis, the preanalytical errors were taken into consideration to avoid false results for pH and ionized calcium (iCa). To achieve correct heparin and blood concentration correct blood volume of sample was collected with immediate mixing after sampling to avoid false low levels of iCa. After collection of the sample the blood was immediately analyzed in the clinical laboratory for ABG (within 20 minutes) to avoid any discrepancy in the results because loss of pCO_2_ from the collected sample may increase pH as alkaline pH increases protein bound calcium and decreases iCa levels. On the other hand acidic pH decreases protein bound calcium and increases iCa levels. Even haemolysis results in false levels of low ionized calcium.

Among the ABG parameters pH, pCO_2_, HCO_3_ and ionized calcium (iCa) were mainly taken into consideration. Ionized calcium levels were calculated both as actually measured levels and corrected mathematically at pH 7.4 to avoid influence of sample handling. Delta ionized calcium was also calculated to move towards more accuracy.

*Calculation of corrected ionized calcium (at pH7.4) and delta ionized calcium*

Following formula was used to calculate corrected ionized calcium [7].

Corrected ionized calcium = Measured ionized calcium × [ 1 – 0.53 × (7.4 – actual pH) ]

Delta ionized calcium is the difference between ionized calcium and calculated ionized calcium at pH 7.4

### Statistical analysis

The statistical analysis was performed using Statistical Package for Social Science program (version 16.0; SPSS Inc., Chicago, IL). In the present observational study 6 to 15 (Average 10) successive readings of ABG were noted for each patient. For the descriptive analysis, continuous variables were described as medians with interquartile ranges (IQRs) and categorical variables as percentages and frequencies. Spearman correlation test was done for calculation of correlation between pH and other ABG parameters. The test with p value < 0.05 was considered statistically significant.

## Results

Demographic and clinical characteristics of SARS-CoV-2 patients are summarized in Table 1. On initial hospital evaluation, median levels of total calcium, total proteins, albumin and NLR ratio was 1.98 mmol/L (1.64-2.34), 67 g/L (58-74), 26 g/L (15.50-35.50) and 8.7 (6.5-29.5) respectively. With median value of 1.98 mmol/L all the patients were found to be hypocalcemic. Normal levels of serum proteins though on lower side were observed with median value 67 g/L.

**Table 1:** Demographic and clinical characteristics of SARS-CoV-2 patients HTN – hypertension; DMT2 – diabetes mellitus type 2; NLR –neutrophil-to-lymphocyte ratio; pCO_2_ – partial pressure of carbon dioxide; HCO_3_ – bicarbonate ion; O_2_sat – oxygen saturation

Parameter	Median (IQR) or N (%)
Age, years (n=17)	62 (31.5–83.5)
Male (n=17)	11 (65%)
Female (n=17)	6 (35%)
With Comorbidities<br>(HTN, T2DM)<br>(T2DM)<br>(HTN)<br>No comorbidity	14 (82%)<br>6 (35%)<br>7 (41%)<br>1 (6%)<br>3 (18%)
Acid Base Imbalance<br>Respiratory Alkalosis<br>Respiratory Acidosis<br>Mixed disorder	<br>7 (41%)<br>1 (6%)<br>9 (53%)
Non survivors<br>(T2DM)<br>(HTN)	7(41%)<br>6 (86%)<br>1 (14%)
Total Calcium, mmol/L (n=17)	1.98 (1.64–2.34)
Total Protein, g/L (n=17)	67 (58–74)
Serum Albumin, g/L (n=17)	26 (15.50–35.50)
NLR (n=17)	8.7 (6.5–29.5)
pH (n=170	7.43 (7.29–7.56)
pCO_2_, mmHg (n=170)	33.6 (14.05–53.97)
HCO_3_, mmol/L (n=170)	23.1(13–32.79)
O_2_, sat % (n=170)	95.3 (85.2–105.2)
Ionized Calcium, mmol/L<br>(n=170)	0.86 (0.05–1.57)

All the patients also showed Hypoalbuminemia with median value of 26 g/L. Whereas, NLR the ratio of absolute count of neutrophil to lymphocyte with median level 8.7 was also calculated as a stress factor in clinical ICU practice.

Among the total critically ill patients 65% were males. Fourteen (82%) out of seventeen patients were having comorbidities i.e. 41% were diabetic, 6% were hypertensive whereas 35% were found to be both diabetic and hypertensive. 18% were found to be having no comorbidity. Out of seventeen patients, seven (41%) could not survive. In non-survivors T2DM (86%) was the most common comorbidity followed by HTN (14%). Initially, for the observations of acid-base imbalance 53% were suffering with mixed disorder of acid-base balance (respiratory alkalosis and metabolic acidosis), 41% with respiratory alkalosis and 6% with respiratory acidosis. After monitoring 6 to 15 successive readings of the ABG samples for each patient we analyzed a total of 170 samples for which median (IQR) was calculated. For pH it was 7.43 (7.29-7.56), for pCO_2_ 33.6 mm/Hg (14.05-53.97), for HCO_3_ and iCa 23.1 mmol/L (13-32.79) and 0.86 mmol/L (0.05-1.57) respectively and for O_2_ saturation 95.3% (85.2-105.2)

Table 2 revealed percentage occurrence of abnormal levels of ABG parameters of SARS-CoV-2 patients in which low levels of ionized calcium was 98% as compared to abnormal levels of pH being 40%, pCO_2_ being 52%, HCO_3_ being 58% and low saturation of oxygen being 46%.

**Table 2:** Percentage occurrence of abnormal levels of ABGparameters in SARS-CoV-2 patients

Parameter	Readings n=170 (%)
pH	68 (40)
pCO_2_	88 (51.8)
HCO_3_	99 (58.2)
O_2_ saturation	78 (45.9)
Ionized Calcium	167 (98.2)

**Figure 1:**
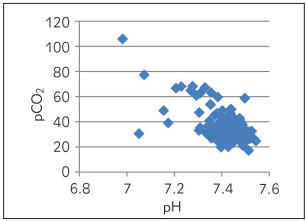
pH vs pCO_2_

**Figure 2:**
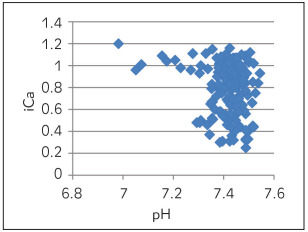
pH vs ionized calcium (iCa)

**Figure 3:**
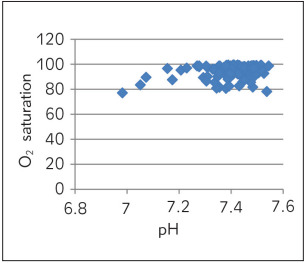
pH vs O_2_ saturation

**Figure 4:**
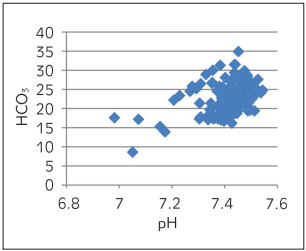
pH vs HCO_3_

**Figure 5:**
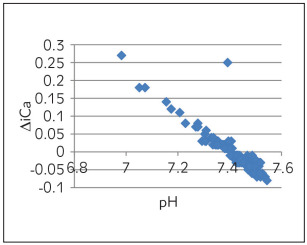
pH vs delta ionized calcium (∆ iCa)

Table 3 depicts Spearman correlation to see the association of pH with other parameters of ABG. A strong negative correlation was observed between pH and pCO_2_ (Figure 1) and a marginal negative correlation were observed between pH and ionized calcium (Figure 2). No association was found between pH and O_2_ saturation (Figure 3). Whereas, a strong positive correlation was observed between pH and HCO_3_ (Figure 4) and between pH and delta ionized calcium (Figure 5). Delta ionized calcium is the difference between ionized calcium and calculated ionized calcium at pH 7.4. Figure 5 shows both positive (for acidic) and negative (for alkalosis) correlation for pH and delta ionized calcium. Since, our number of cases was mostly of alkalosis (41%) and mixed disorder (53%) as compared to acidosis (6%) the graph depicts more of negative correlation.

**Table 3:** Spearman correlation of pH with pCO_2_, HCO_3_,ionized calcium and O_2_ sat in SARS-CoV-2 patients *p<0.05 considered statistically significant<br>r_s_ – Spearman correlation

Parameter	Patients with SARS-CoV-2 pH	
	r_s_	p value
pCO_2_	-0.335	< 0.001*
HCO_3 _	0.399	<0.001*
Ionized Calcium	-0.151	0.048*
O_2_ saturation	0.015	0.847
Delta ionized calcium	0.213	0.005*

## Discussion

A low level of calcium is a common laboratory abnormality in viral infection and pneumonia [8]. Previous studies reported that calcium played a vital role in viral infections and replicative mechanisms of SARS-CoV, MERS-CoV and Ebola virus. Calcium ions directly interacted with fusion peptides of these viruses promoting their replication [9] [10]. Hypocalcemia is a common phenomenon among critically ill patients, its prevalence ranges from 15% to 88% in adults [11]. Similar to recent study by Zhou et al. [12] our study revealed hypocalcemia in all cases regardless of severity of their condition in the early stage of viral infection. Recently, Filippo et al. [13] observed 80% hypocalcemic patients in their study.

Considering age factor 59% of our patients were older than 60 years. Median age of the studied patients was 62 years old which suggested that aged people were more susceptible to severe COVID-19. In another study by Liu et al. [14] 63.6% of their COVID-19 patients were also of older age (>65 years). Age factor does affect the recovery capacity of a person at the later stages of infection because the liver and kidney function of an individual declines as age advances leading to decrease in intestinal calcium absorption due to low levels of 25-hydroxy cholecalciferol. Similarly, we observed in our study, that the severe critically ill patients who could not survive were older (>60 years) in age.

In our present study among the non-survivors T2DM (86%) was the most common comorbidity. In Diabetes Mellitus secretion of Parathyroid Hormone (PTH) declines, resulting in decrease disrupted calcium homeostasis that is cellular calcium depletion occurs. PTH stimulates calcitriol (1,25 dihydroxy cholecalciferol), which regulates calcium homeostasis in the body. Calcitriol induced Ca^2+^ signals (oscillations) regulate insulin secretion from the pancreatic cells [15] [16]. Thus, the rapid increase in intracellular Ca^2+^ triggers insulin release.

The perception that acid base imbalance in diabetes is confined to metabolic acidosis is also challenged by our results. The most common disturbance observed was mixed disorder of acid-base balance present in 9 patients. Respiratory disturbance predominantly respiratory alkalosis was present in 7 patients whereas, only 1 patient suffered with respiratory acidosis.

Calcium is predominantly bound to albumin. Almost 30-55% in the plasma and a decrease in serum albumin will also cause hypocalcemia. Ionized calcium binds to negatively charged sites on protein molecules. Therefore, Hypoalbuminemia is associated with Pseudohypocalcemia which is reduction in total calcium concentration even though there are normal iCa levels [17].

To sustain normal organ function calcium homeostasis has to be maintained. Changes in intracellular calcium homeostasis can also promote the activation of inflammatory pathways leading to increase in tumor necrosis factor (TNF) and IL-6 [18] [19]. In addition, hypoxia of tissue and organ induces cell membrane damage resulting in calcium influx [14].

Chernow et al. [20] in their study demonstrated that hypocalcemia was associated with prolonged stay in ICU and increased mortality, similar to our study, in which 41% were non-survivors, with stay in ICU more than 5 days. Moreover, poor prognosis observed in hypocalcemic patients with severe SARS-CoV-2 was similar to many recent studies [14]. Similar to our observations, some authors also identified hypocalcemia as a relevant and independent risk factor for worse clinical outcome, associated with higher mortality in hospitalized and critically ill patients admitted in ICU [14] [21] [22] [23]. Though our results showed marginal significant correlation of pH and iCa as such but strong correlation between pH and delta iCa cannot be ignored too. Moreover, 98% prevalence of low levels of iCa as compared to other parameters of ABG is also worth mentioning.

## Limitation

The main limitation of our study is that the sample size was relatively small. We could only proceed with the samples from patients admitted from October to December. Larger studies are needed to confirm our findings. Possibility of bacterial-infection also could not be ruled out because of high NLR ratio in these studied SARS-CoV-2 patients. We consider that additional studies are required to support these findings.

## Conclusion

Our study suggests that ABG analysis acts as a momentous indicator for critically ill patients admitted in Intensive Care Unit (ICU). Estimation of iCa in this critical care setting acts as a distinctive biochemical feature of SARS-CoV-2 disease, as an initial assessment tool, for hypocalcemia, has a potential impact on its severity. It is also suggested that ionized calcium-the physiologically active component of total calcium should be preferred as routine method for determining the level of calcium in all patients. Moreover, iCa is also easy to measure (within 15 to 20 minutes) making clinicians in identifying severe patients at initial hospital valuation. Hypocalcemia represents a novel prospective treatment goal worth to be treated at the earliest for improvements in patient care.

## Dodatak

### *Acknowledgments*.

The authors are grateful toProfessor Malkinder Singh, Department of English of Khalsa College Amritsar for vetting the paper withrespect to grammatical mistakes.

### Conflict of interest statement

All the authors declare that they have no conflictof interest in this work.
